# Incidental Prenatal Diagnosis of Sex Chromosome Aneuploidies: Health, Behavior, and Fertility

**DOI:** 10.5402/2011/807106

**Published:** 2011-12-12

**Authors:** J. J. P. M. Pieters, A. J. A. Kooper, A. Geurts van Kessel, D. D. M. Braat, A. P. T. Smits

**Affiliations:** ^1^Department of Obstetrics and Gynaecology, Radboud University Nijmegen Medical Centre, 6500 HB Nijmegen, The Netherlands; ^2^Department of Human Genetics, Radboud University Nijmegen Medical Centre, 6525 GA Nijmegen, The Netherlands

## Abstract

*Objective*. To assess the diagnostic relevance of incidental prenatal findings of sex chromosome aneuploidies. *Methods*. We searched with medical subject headings (MeSHs) and keywords in Medline and the Cochrane Library and systematically screened publications on postnatally diagnosed sex chromosomal aneuploidies from 2006 to 2011 as well as publications on incidentally prenatally diagnosed sex chromosomal aneuploidies from 1980 to 2011. *Results*. Postnatally diagnosed sex chromosomal aneuploidies demonstrated three clinical relevant domains of abnormality: physical (22–100%), behavior (0–56%), and reproductive health (47–100%), while incidentally prenatally diagnosed sex chromosomal aneuploidies demonstrated, respectively, 0–33%, 0–40%, and 0–36%. *Conclusion*. In the literature incidental prenatal diagnosis of sex chromosomal aneuploidies is associated with normal to mildly affected phenotypes. This contrasts sharply with those of postnatally diagnosed sex chromosomal aneuploidies and highlights the importance of this ascertainment bias towards the prognostic value of diagnosis of fetal sex chromosomal aneuploidies. This observation should be taken into account, especially when considering excluding the sex chromosomes in invasive prenatal testing using Rapid Aneuploidy Detection.

## 1. Introduction

Sex chromosomal aneuploidies (SCAs) are usually diagnosed postnatally in association with specific physical, developmental, and psychological health problems, diminished fertility, or infertility (incidence 1 in 400) [1]. Prenatally, the overall incidence of SCAs is 1 in 435 [2], depending on the reason for referral for invasive prenatal testing. For pregnant women whose unborn children are at risk for Down syndrome (e.g., maternal age >35 years or a positive first trimester screening result without ultrasound abnormalities), the incidence of SCAs is comparable to that of Down syndrome (1 in 300) [3], which represents 25% of all abnormal karyotypes identified [4]. Diagnoses of SCAs in routine prenatal invasive testing are often incidental and present unforeseen findings to the parents, whereas their diagnostic significance is uncertain [5]. As such, these SCAs may be considered as nondiscretionary test information. They are indicative of an abnormality that in the end may not cause any relevant symptoms or only mild symptoms such as a slightly reduced intelligence and/or a diminished fertility. Taking into account that parents are primarily tested to exclude Down syndrome, the diagnosis of an SCA may elicit unforeseen dilemmas about whether or not to terminate the pregnancy [6]. A recent study revealed that in 36% of prenatally detected sex chromosome trisomies these findings have resulted in pregnancy termination [7]. Early and presymptomatic diagnoses may, however, provide opportunities for early treatment of certain health and developmental problems and, as such, may represent an advantageous step towards future healthcare for the child. This latter issue has amply been discussed in the literature [8–12]. 

Since molecular diagnostic technologies based on Multiplex Ligation-dependent Probe Amplification (MLPA) or Quantitative Fluorescent Polymerase Chain Reaction (QF-PCR) are designed for rapid aneuploidy detection (RAD) of the most common aneuploidies (i.e., of chromosomes 13, 18, 21, X, and Y) [13], the question of whether or not to continue the inclusion of probes for the sex chromosomes arises, since sex chromosome detection does not always provide useful information to parents referred for advanced maternal age [14]. Replacement of full karyotyping with stand-alone RAD for chromosomes 13, 18, and 21 only, would avoid incidental SCA findings. An advantage of early SCA detection may provide opportunities for preventive programs aimed at ameliorating the quality-of-life. This study consists of a systematic literature review assessing the syndrome-specific, health-related, quality of life (QOL) aspects of patients with postnatally diagnosed SCAs and comparing these results with those of prenatally incidentally diagnosed SCAs.

## 2. Methods: Sources, Search Strategy, and Study Selection

### 2.1. Sources and Search Strategy

We performed systematic electronic searches during the period of August 2010 until March 2011 in Medline (PubMed) and the Cochrane Library (Database of Systematic Reviews and the Cochrane Pregnancy and Childbirth Group's Trials Register) using medical subject headings (MeSHs), filters, and keywords. We employed a two-stage process for our searches: first, we assessed the literature over the last 5 years (2006–2011) aimed at gathering information about specific phenotypes associated with postnatally diagnosed SCAs. Second, we assessed the literature from 1980 onward aimed at gathering information about the difference in phenotype after an incidental prenatal detection of SCAs and the associated phenotypes that were ascertained postnatally.

### 2.2. Study Selection

We used the following terms in our PubMed literature search: sex chromosome aberrations “or” sex chromosome disorders “or” X chromosome “or” chromosome human X “or” Y chromosome “or” Turner syndrome “or” 45,X syndrome “or” Klinefelter syndrome “or” 47,XXY syndrome “or” Triple X syndrome “or” Trisomy X syndrome “or” 47,XXX syndrome, and “not” Fragile X syndrome. We used filters for publications in English dealing with human subjects.

We prepared a PICO research query in order to focus our search on the period 1980–2011.

**PICO Research Query Used to Focus Literature Search and Data Abstraction**

How does a prenatal diagnosis of SCA affect quality of life for individuals, whether intended or not? PICO research query:

P: fetus/child with SCA,
I: invasive prenatal diagnostic test with *incidental* finding of fetal SCA,
C: prenatal ultrasound abnormality or postnatal diagnosis SCA,
O: postnatal prognosis in syndrome-specific health-related quality of life.

The accountability for this research period is the fact that the first study on ultrasonographically abnormal fetuses were published around 1980. We double checked all retrieved publications for the following MeSH inclusion criteria and keywords: prenatal diagnosis, disease, prognosis, quality of life, postnatal, phenotypic, prospect, prognosis, unforeseen, unintended, and accidental. We combined all search terms and keywords with (MeSH, tiab).

#### 2.2.1. Inclusion Criteria (Stage 1)

All publications used were published in peer-reviewed journals listed in the Science Citation Index. We included original articles, reviews, randomized trials, cohort studies, case-control studies, case reports, letters, expert opinions, and consensus meeting reports dealing with either full-blown or mosaic SCA.

#### 2.2.2. Inclusion Criteria for Publications according to the Research Query (Stage 2)

We used the criteria of stage 1, publications concerning the incidental prenatal SCA findings in relation to the postnatal, syndrome-specific, QOL prognosis and a comparison with this prognosis for postnatally ascertained SCAs.

#### 2.2.3. Exclusion Criteria

We excluded all publications dealing with ultrasonographic fetal abnormalities, epidemiology, registration, or counseling issues in relation to SCAs, cytogenetic, or molecular technical prenatal diagnostic research and animal studies.

#### 2.2.4. Publication Selection for Stages 1 and 2

In both stages, the authors (J. J. P. M. Pieters and A. J. A. Kooper) screened the titles of all potentially relevant publications and marked them as “included” (title clearly related to SCA and meeting the inclusion criteria set for both stages) or “excluded” (not related, other syndrome, or not meeting the inclusion criteria). Next, the authors (J. J. P. M. Pieters and A. P. T. Smits) screened the abstracts of the remaining titles, again marking them as “included” or “excluded.” All authors discussed the items that were unclear until they reached agreement. Finally, we grouped all included publications by syndrome.

## 3. Results

### 3.1. Stage 1: SCA and Syndrome-Specific Quality of Life (2006–2011)

Using the criteria set (see Methods) we retrieved 1093 potentially relevant publications from the period 2006–2011. Specifically, we screened for issues concerning syndrome-specific, health-related, QOL prognoses for persons exhibiting SCA. This resulted in 607 selected publications dealing with Turner syndrome (TS, *n* = 400), Klinefelter syndrome (KS, *n* = 193), Triple X syndrome (Tr X, *n* = 7), or other SCAs (*n* = 7); see Table 2.

### 3.2. Syndrome-Specific, Health-Related, Quality-of-Life Domains

Based on the results, we were able to determine three domains that could be considered the core outcome domains in assessing the syndrome-specific QOL of patients with SCA, that is, physical health, behaviour, and reproductive health (Tables 1 and 2).

#### 3.2.1. Domain 1: Physical Health

Physical health was characterized by abnormal growth, diminished bone mineral density, cardiovascular, metabolic, and other diseases, autoimmune disease, increased cancer risk, dental problems, otologic problems, and overall disease susceptibility and mortality. Also, early screening and preventive programs for health were mentioned. All SCA-related health hazards and overall QOL were said to benefit from early recognition, prompt diagnosis, and pharmacologic or psychosocial treatment in an early stage. Other SCAs were associated with the exacerbation of certain medical conditions relative to the burden of these conditions among the general population. Examples of such conditions are asthma, congenital heart defects, and increased morbidity or mortality rate caused by epileptic insults, cardiovascular disease, or respiratory disease. Most health hazards were described in association with TS and KS, while Tr X syndrome and other SCA-related disorders were only rarely discussed in relation to domain 1. A relevant finding was the importance of early recognition and preventive treatment of certain health problems.

#### 3.2.2. Domain 2: Behavior

Problematic psychosocial functioning and learning capacities, diminished QOL, and problems with relationships and sexuality were discussed in association with SCA. Certain behavioral weaknesses (Diagnostic and Statistical Manual of Mental Disorders (DSM) IV: Adjustment Disorder with Mixed Disturbance of Emotions and Conduct) and limited skills associated with specific brain asymmetries, especially in TS and KS, have been found, and a high body mass index has been related to a negative body attitude. Early psychoeducational support and medical therapy were found to positively affect the patients concerned. Health aspects, infertility, aberrant stature, and psychosocial disabilities negatively influenced the QOL. Positive effects of early growth hormone and estrogen treatment on the psychosocial functioning and self-esteem of girls with TS and of testosterone treatment on the pubertal changes, behavior, and sexuality of boys with KS have been reported. Domain 2 was a focal point of attention both for individuals with Tr X syndrome and those with other SCAs. Attention deficit disorders, autism spectrum disorders, mood disorders, and tic disorders were found.

#### 3.2.3. Domain 3: Reproductive Health

A variety of aspects of fertility and endocrinology were denoted as problematic in TS and KS, including problematic puberty or abnormal gonadal development (TS: *n* = 25 and KS: *n* = 33 reports, resp.), but there were very little reports of infertility associated with other SCAs. Although assisted reproduction techniques (ART) created new possibilities of procreation, associated risks were mentioned.

Most patients with a nonmosaic SCA karyotype (TS and KS) were infertile, whereas those with mosaicisms were usually fertile. Also here, early gamete cryopreservation and ART have made procreation possible, but medical hazards and the risk of transmission of aneuploidy to the next generation have been discussed.

**Figure 1:**
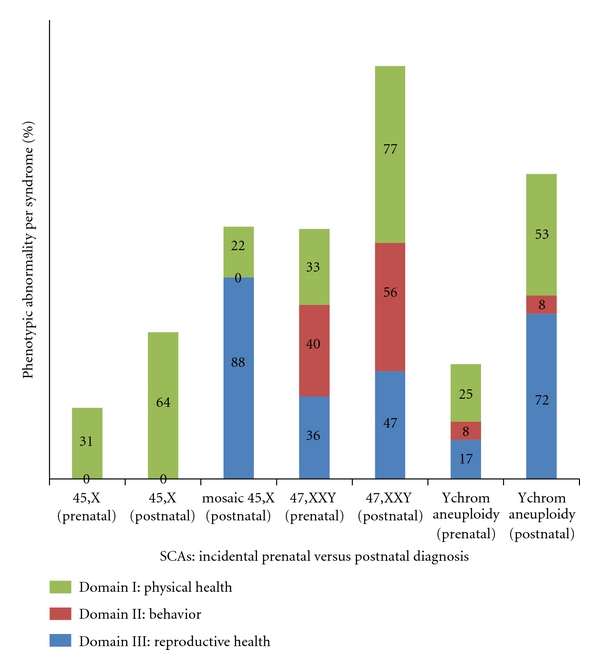
Publications on SCA, 1980–2011, syndrome-specific quality-of-life domains incidental prenatal detection versus postnatal diagnosis.

**Figure 2:**
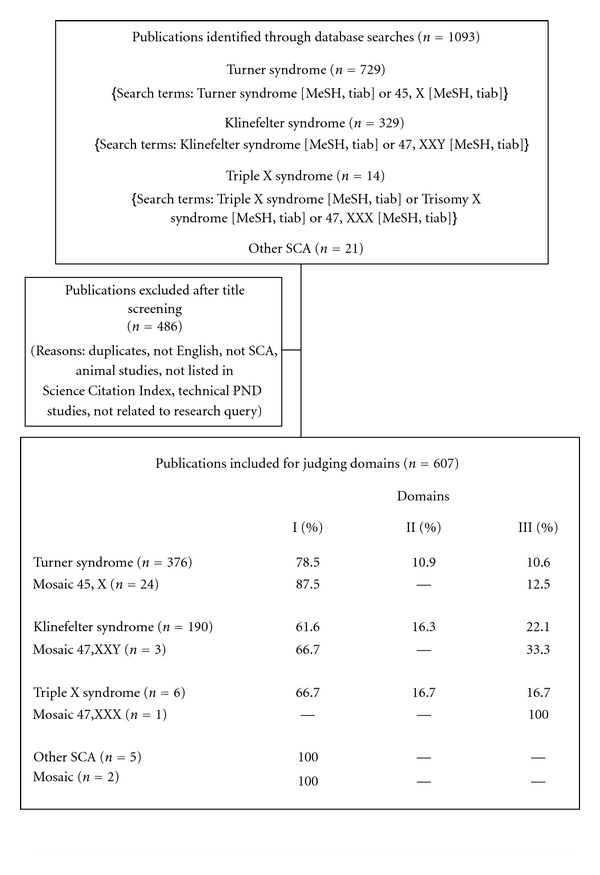
Prisma Flowchart of systematic study selection (stage 1: 2006–2011).

Domain 1 (physical health) was mostly discussed in the SCA literature in general and, specifically, in conjunction with all types of SCA. Behavior was not a prominent issue, and reproductive health was mostly discussed in relation to KS. For further details about the incidence of the domains discussed per syndrome in the period 2006–2011, see Table 2 and Figure 1; the study selection process is shown in Figure 2.

### 3.3. Stage 2: Incidental Prenatally Diagnosed SCA and Postnatally Ascertained SCA, 1980–2011

**Figure 3:**
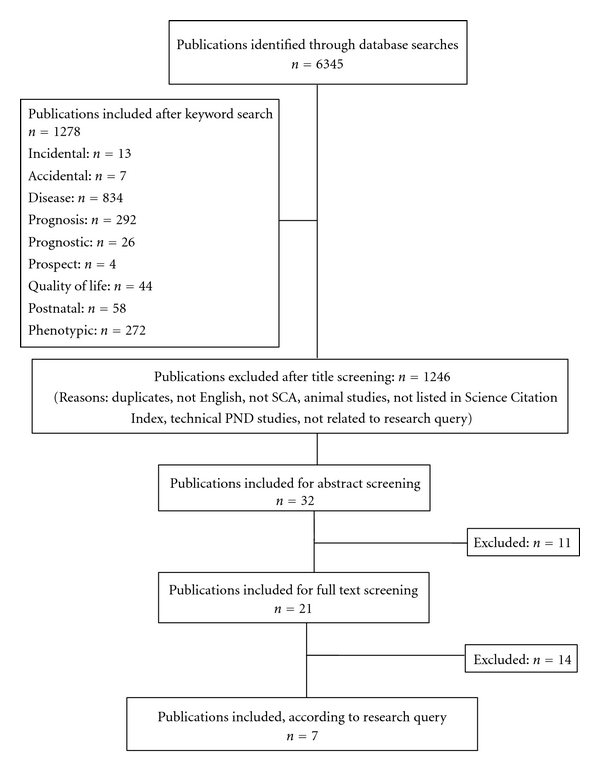
Flow diagram of systematic literature search of SCA, 1980–2011: phenotype and health of incidental prenatal diagnosis versus postnatal diagnosis.

We searched PubMed and the Cochrane Library for eligible publications about the incidental prenatal diagnosis of SCA with special attention to our PICO research query. We identified 6345 potentially relevant SCA publications using the MeSH terms, search conditions, and filters described in the Methods section. We excluded publications that did not meet all our inclusion criteria. We included 1278 publications that were subjected to peer review with regard to the relevance of an incidental intrauterine SCA finding for the long-term developmental prognosis. Finally, we included 32 publications for abstract screening and categorization by domain. After abstract screening, 21 potentially relevant publications were kept, seven of which were selected after full text screening (Table 3 and Figure 3) relating to the PICO query: an incidental prenatal diagnosis of SCAs, description of the associated phenotype and the syndrome-specific quality-of-life aspects for postnatal life, and a comparison with postnatally ascertained SCAs.

(1) Wheeler et al. [15] compared the postnatal phenotypes of six incidental prenatally diagnosed 45,X/46,XY fetuses of couples with an increased risk of a chromosome abnormality. The postnatally diagnosed children were all phenotypically abnormal in domain 3, whereas the incidentally prenatally diagnosed fetuses all developed into phenotypically normal boys.

(2) Pettenati et al. [16] reported a comparison of three incidental prenatally diagnosed cases with 45,X/47,XYY mosaicisms with four cases diagnosed postnatally with similar mosaicisms. The three incidental prenatally diagnosed cases were all physically normal at birth, whereas the postnatally diagnosed cases all exhibited phenotypic anomalies (which had been the reason for karyotyping). 

(3) Hsu [17] reviewed many studies on Y chromosome aneuploidies (except nonmosaic 47,XYY), going back to publications of as early as 1961 and reported in this elaborate review on 600 cases with mosaicisms for genotype-phenotype correlations. Of these, 93 were incidentally prenatally diagnosed, while all the other cases were postnatally diagnosed because of phenotypic anomalies. All postnatally diagnosed cases were phenotypically abnormal (which had been the reason for karyotyping), while 67–97% of the prenatally diagnosed cases exhibited a normal male phenotype at birth.

(4) Koeberl et al. [18] reported on 12 incidental prenatally diagnosed 45,X/46,XX patients and compared the outcome with 41 postnatally diagnosed girls. They concluded that, although a certain ascertainment bias did exist, the prevalence of a 45,X/46,XX mosaicism was 10 times higher among the group diagnosed prenatally as compared to postnatally diagnosed TS patients. Absence of hydrops fetalis may account for the milder phenotype of the prenatally diagnosed group.

(5) Gunther et al. [19] compared 16 incidental prenatally diagnosed patients with TS with 72 traditionally diagnosed children (typical fetal anomalies on ultrasound or postnatal clinical features). The incidental group exhibited significantly fewer phenotypic TS features than the traditional group. The authors concluded that a significant ascertainment bias does exist in our understanding of TS, with important implications for prenatal counseling in case no fetal abnormalities are found.

(6) Zeger et al. [20] compared phenotype data from 55 postnatally diagnosed 47,XXY boys with those from 35 prenatally diagnosed 47,XXY boys and found no significant differences between the two groups. Invariably, all features occurred comparable in both groups.

(7) Girardin et al. [21] reported a comparison of clinical symptoms of adolescents with KS after incidental prenatal diagnosis (*n* = 11) with those of adolescents diagnosed because of an abnormal phenotype (*n* = 17). They concluded that there were some differences between the two groups with respect to the presence of gynaecomastia, school delay, and testosterone substitution. Although these differences were not significant, the incidence of phenotypic problems in the postnatally diagnosed group was somewhat higher than in the incidental prenatally diagnosed group. The incidences differed significantly in comparison with the general population.

### 3.4. Conclusions of Stage 2

Physical health (domain 1) was abnormal for 0–33% of those who were diagnosed incidentally prenatally, whereas it was abnormal for 22–100% of those who were diagnosed postnatally. The behavior (domain 2) was abnormal for 0–40% of the incidental prenatally diagnosed patients, while the behavior was abnormal for 0–56% of the postnatally diagnosed patients. The reproductive health (domain 3) was problematic for 0–36% of the incidental prenatally diagnosed patients, whereas it was problematic for 47–100% of the postnatally diagnosed patients (Table 3, Figure 3).

Four of seven publications regarding the incidental prenatal diagnosis of SCAs dealt with mosaic SCAs, which resulted in normality in the domains of physical health (100%), behavior (100%), and reproductive health (67–100%). For nonmosaic cases, the normality results were: physical health, 67–75%; behavior, 60–100%; reproductive health, 64–83%. For postnatally ascertained SCAs, the mosaicism-related normality results were: physical health, 0–47%; behavior, 75–100%; reproductive health, 0–34%, while for nonmosaic cases, the normality results were: physical health, 0–36%; behavior, 0–44%; reproductive health, 0–53%.

## 4. Discussion

A putative change in prenatal diagnosis policy from full karyotyping to stand-alone, rapid aneuploidy detection (RAD) with the standard inclusion of probes for the sex chromosomes needs to be assessed carefully. The purpose of this paper was to reveal the clinical relevance of a diagnosis of SCA for the postnatal quality of life (QOL) of symptomatic individuals and for those in whom the prenatal diagnosis was an incidental finding. First, we assessed the SCA literature of the last 5 years, in order to gather information about the specific, phenotypic, and clinical problems associated with postnatal SCA detection. By doing so, we found that these publications all addressed one or more domains of syndrome-specific health. Next, we assessed whether the phenotypes of patients with SCA were comparable, irrespective whether the diagnoses were made postnatally or prenatally without any ultrasonographic abnormalities, due to an incidental finding. To this end, we screened all SCA literature between 1980 and 2011. We used 1980 as a starting point since the first studies of fetal ultrasound abnormalities that were associated with a genetic syndrome were published around that time [22].

We found in our first literature search that physical health (domain 1) is by far most often discussed (74%), followed by reproductive health problems (domain 3, 14%) and behavior (domain 2, 12%); see Tables 1 and 2. Problematic physical health is an important issue for patients with an SCA. It manifests itself as disturbances in growth or bone mineral density and cardiac, autoimmune, and other diseases, and it certainly influences the QOL. Many publications dealing with domain 1 mentioned possible early preventive measures. Timely screening and treatment may significantly improve the QOL of patients with certain very health-menacing conditions such as abnormal growth, diminished bone mineral density, and cardiovascular problems. Early detection of SCA-related problems varied between domains. Some authors have elaborated on the minimal negative side effects of lifelong hormonal treatment, but it is clear that the positive effects of this treatment far exceed the potentially negative effects [23, 24]. Apparently, reproductive health problems and behavioral abnormalities trouble these patients to a much lesser extent than physical health problems. Early preventive management has been described; for example, induction of puberty and breast development in TS and technical advances in ART increasingly permit parenthood. We are aware of the fact that the number of selected publications is not automatically a measure of its importance, but its frequency is of note. 

In the second stage of our literature study, we focused on the impact of the *incidental* finding of a prenatal SCA on the postnatal outcome. The selected publications report on comparisons of patients who were incidentally prenatally diagnosed with patients who were diagnosed through traditional postnatal karyotyping (either nonmosaic or mosaic). In six out of seven publications, the overall phenotypic outcomes affected incidentally prenatally diagnosed SCA patients significantly less than those who were postnatally diagnosed and karyotyped because of phenotypic abnormalities. In one publication [20], no significant differences in certain phenotypic characteristics were noted after prenatal or postnatal diagnosis.

 It is well known that certain fetal ultrasonographic abnormalities are associated with a poor prognosis for the child after birth, because they reflect early disturbances in organ functioning [18]. Genetic mosaicisms are known to be related to mild or even complete lack of phenotypic features, and those affected may go through life without ever knowing they carry a genetic abnormality [21, 25]. Saenger's review [26] showed that most prenatal diagnoses of TS occur by chance after routine invasive procedures for advanced maternal age, and the phenotypes of these women are usually less pronounced [19]. Those with TS detected by ultrasound (increased nuchal translucency or fetal hydrops) exhibit a high rate of spontaneous fetal loss or associated cardiac or renal disease [18, 27]. Bondy [10] discusses the fact that incidentally diagnosed TS in prenatal diagnostic tests for advanced maternal age lead to termination of most of these pregnancies, and that the ability to assess clinical outcome is limited. High termination rates for incidentally diagnosed TS reflect overrated pessimistic views, as the phenotype of someone with an incidentally ascertained TS is compared with the phenotypic problems of those who are postnatally diagnosed, or prenatally diagnosed because of fetal abnormalities. Only 10% of those with KS are diagnosed prenatally, another 25% are diagnosed during childhood or adolescence, and 65% remain undiagnosed [28]. KS is the most frequent genetic cause of infertility [29, 30] (11% of azoospermic men), and parents often report frustration with the consequences of a delay in diagnosis [31].

Publications on the diagnosis of Tr X syndrome did not address its incidental prenatal finding and the subsequent clinical consequences. A recent literature review of Tr X [32] reports mainly personality and behavioral problems and finds that many of the studies reviewed were “biased because of referral bias.” Furthermore, the incidental prenatal finding of Tr X is discussed only in terms of termination rates and intercultural differences. The author concludes that Tr X syndrome is not rare, but often remains undiagnosed. Approximately 20% of other SCAs have higher-grade chromosome aneuploidies (e.g., 48,XXXY) or mosaicisms and the associated clinical health issues are usually very mild.

To the best of our knowledge, this study for the first time categorizes the abundant SCA literature into three syndrome-specific, QOL-related domains of health. This categorization provides a way to judge the importance of phenotypic problems of an incidental prenatal ascertainment of SCA relative to a postnatal ascertainment of SCA. However, we acknowledge that even if there is an abundance of publications about the clinical implications of SCA for the affected individual, there is little literature that compares the difference in QOL prognoses for incidental prenatally diagnosed patients to those diagnosed postnatally. Despite this limitation, however, we have endeavored to review the literature in a way that is helpful to professionals who wish to use this information in counseling and decision-making processes.

Routine prenatal testing procedures with the standard diagnostic outcome of fetal gender offer a unique opportunity for early prevention and management of SCA-related disease, psychological issues, and fertility issues. This benefit is evident from the professional point of view, but it should be balanced against the possible negative impact of this unexpected diagnosis for the future child. Incidental prenatal diagnosis of an SCA may cause stigmatization and possible damage to the child's self-esteem or distortion of the family's perception of the child. Indeed, parents may consider termination of the pregnancy while being uncertain about the prognosis for the child [33, 34]. Molecularly targeted testing through RAD enables the exclusion of the sex chromosomal markers, thus avoiding unexpected sex chromosome-related SCAs. A discussion about this policy has been published [35]. It is considered a more or less acquired right in routine prenatal practice that pregnant women can be informed about the sex of their unborn children if they wish to know. However, ultrasonography can also determine fetal gender with high accuracy (98.3%) at 13 weeks of gestation [36], and an almost 100% accuracy is achieved around 20 weeks of gestation [37].

## 5. Conclusion

Early knowledge of an SCA could help the parents and their child to adapt to the consequences of the corresponding syndrome in a timely fashion rather than having the information presented at puberty or even a later stage in life. This fact must be balanced against the ethical concerns about the diagnosis of a genetic condition with an uncertain prognosis. This paper shows that, in the medical literature, the syndrome-specific QOL of patients with an SCA contrasts sharply between postnatal and incidental prenatal diagnoses for the three major domains of health. Phenotypic abnormalities in prenatal or postnatal life were found to be associated with significantly more severe clinical consequences. In contrast, the absence of fetal abnormalities in incidental prenatal diagnoses was found to be associated with a normal to mildly affected phenotype, and, as such, a significant ascertainment bias may exist in our understanding of SCA. Although it seems rather obvious that the outcome of a child with an incidental discovery of a sex chromosome abnormality is better than one who is diagnosed postnatally, this review of the literature since 1980 shows that counselors may reassure the parents in the knowledge that indeed all studies until now support this conclusion.

## Figures and Tables

**Table 1:** Categorization of disease-specific QOL domains related to SCA.

Domain I: physical health	Growth and bone mineral density Cardiovascular, metabolic, and other disease Autoimmune disease Other SCA-associated health problems Overall disease susceptibility and mortality

Domain II: behavior	Psychosocial functioning Quality of life Sexuality

Domain III: reproductive health	Puberty Fertility Assisted reproduction techniques Disease

**Table 2:** Number of publications on SCA in the period 2006–2011, syndrome-specific quality-of-life domains.

Publications per domain	45,X (*n*,%)	45,X mosaic (*n*,%)	47,XXY (*n*,%)	47,XXY mosaic (*n*,%)	47,XXX (*n*,%)	47,XXX mosaic (*n*,%)	Other SCA (*n*,%)	Other SCA mosaic (*n*,%)
I: physical health (*n* = 446)	295 (78.5%)	21 (87.5%)	117 (61.6%)	2 (66.7%)	4 (66.7%)	—	5 (100%)	2 (100%)
II: behavior (*n* = 73)	41 (10.9%)	—	31 (16.3%)	—	1 (16.7%)	—	—	—
III: reproductive health (*n* = 88)	40 (10.6%)	3 (12.5%)	42 (22.1%)	1 (33.3%)	1 (16.7%)	1 (100%)	—	—

*N* = 607	376 (100%)	24	190 (100%)	3	6 (100%)	1	5 (100%)	2

*N*: number of publications found in the period 2006–2011.

(%): percentage of total number of publications on that type of SCA.

**Table 3:** Publications on the incidental prenatal diagnosis versus postnatal diagnosis of SCA in period 1980–2011. Case-control studies, clinical comparison, and outcome data.

Author, year	*Prenatal incidental* diagnosis SCA outcome of domains	*Postnatal* diagnosis SCA outcome of domains	Type SCA
(1) Wheeler et al., 1988 [15]	6 pregnancies, 3 term, 1 premature delivered infants, 1 termination, 1 intrauterine death: 4 healthy children, 2 fetuses normal on autopsy *Domain I*: 100% normal *Domain II*: 100% no mental retardation *Domain III*: 100% Normal reproductive fertility and normal genitalia	9 children with abnormal intern/extern genitalia, 7 children with ambiguous genitalia at birth, 2 children with primary amenorrhea at age 17 *Domain I*: 22% short stature, webbed neck *Domain II*: 100% no mental retardation *Domain III*: 88%: ambiguous genitalia, changed sexual assignment, rudimentary phallus, urogenital sinus, hypospadias, undescended testes; 22%: primary amenorrhea	45,X/46,XY mosaicism

(2) Pettenati et al., 1991 [16]	3 prenatally detected cases; clinical comparison with the postnatally detected cases *Domain I*: Two phenotypically normal term born infants, 1 posttermination normal male fetus on autopsy *Domain II*: Normal *Domain III*: Normal external male genitalia in both children; fetus with normal position of testes, normal penis, and scrotal development	4 postnatally detected cases, clinical comparison with the prenatally detected cases *Domain I*: All had phenotypic abnormalities: short statue, short limbs, cubitus valgus, nevi, epicanthical folds, depressed nasal bridge, micrognathia, low hair implantation, webbed neck, shield chest, posteriorly rotated ears *Domain II*: One child developmental delay *Domain III*: All children had genital abnormalities: ambiguous genitalia, mixed gonadal dysgenesis, streak gonads, hypospadias, small penile length	45,X/47,XYY mosaicism

(3) Hsu, 1994 [17]	Phenotype of 93 prenatally diagnosed cases, liveborn and abortuses *Domain I*: Not discussed *Domain II*: Not discussed *Domain III*: 67–97% normal gonads or genitalia	phenotype of 503 postnatally diagnosed cases *Domain I*: 0–25% phenotypically abnormal stature *Domain II*: Not discussed *Domain III*: 66–100% abnormal gonads or genitalia	Y chromosome aneuploidy (except nonmosaic 47,XYY)

(4) Koeberl et al., 1995 [18]	12 prenatally diagnosed cases *Domain I*: All (100%): normal growth, 3 (25%) health problems: ASD, ptosis, dysplastic kidneys *Domain II*: 8% mental retardation *Domain III*: 10 (83%): normal; 2 (17%): labial fusion, urogenital sinus	41 postnatally diagnosed patients *Domain I*: 22–53% malformations or phenotypic problems (edema, cardiac, renal otologic, gastrointestinal) *Domain II*: 8% developmental delay *Domain III*: 72% no spontaneous menarche	45,X/46,XX mosaicism

(5) Gunther et al., 2004 [19]	16 incidentally diagnosed cases *Domain I*: 31% heart defects, 25% renal anomalies, length/height deficit (−1.1 SDS), weight deficit (−1.0 SDS) *Domains II* and III: Not discussed	72 traditionally postnatal diagnosed cases *Domain I*: 64% heart defects,19% renal anomalies, length/height deficit (−1.7 SDS), weight deficit (−1.7 SDS) *Domains II* and *III*: Not discussed	45,X

(6) Zeger et al., 2008 [20]	35 prenatally diagnosed boys *Domain I*: Tall stature, hypotonia, increased BMI *Domain II*: Speech and reading therapy *Domain III*: Below average size penis and testes, low testosterone level, low inhibin B and AMH levels, elevated FSH and LH levels	20 postnatally diagnosed boys *Domain I*: tall stature, hypotonia, increased BMI *Domain II*: Speech and reading therapy *Domain III*: Below average size penis and testes, low testosterone level, low inhibin B and AMH levels, elevated FSH and LH levels no significant differences with prenatal group	47,XXY

(7) Girardin et al., 2009 [21]	11 prenatally diagnosed patients *Domain I*: Gynaecomastia 33%, BMI, height: normal *Domain II*: School delay 40% *Domain III*: All had spontaneous puberty, testosterone substitution 36%	17 postnatally diagnosed patients *Domain I*: Gynaecomastia 77%, BMI, height: normal *Domain II*: School delay 56% *Domain III*: All had spontaneous puberty, testosterone substitution 47%	47,XXY
